# Cosmogenic in situ ^14^C-^10^Be reveals abrupt Late Holocene soil loss in the Andean Altiplano

**DOI:** 10.1038/s41467-021-22825-6

**Published:** 2021-05-05

**Authors:** Kristina Hippe, John D. Jansen, Daniel Søndergaard Skov, Maarten Lupker, Susan Ivy-Ochs, Florian Kober, Gerold Zeilinger, José Mariano Capriles, Marcus Christl, Colin Maden, Christof Vockenhuber, David Lundbek Egholm

**Affiliations:** 1 Laboratory of Ion Beam Physics, ETH Zürich, Zürich, Switzerland; 2 Institute of Geological Sciences, Freie Universität Berlin, Berlin, Germany; 3 GFÚ Institute of Geophysics, Czech Academy of Sciences, Prague, Czechia; 4 Department of Geoscience, Aarhus University, Aarhus, Denmark; 5 Geological Institute, ETH Zürich, Zürich, Switzerland; 6 National Cooperative for the Disposal of Radioactive Waste (NAGRA), Wettingen, Switzerland; 7 Institute of Geosciences, University of Potsdam, Potsdam, Germany; 8 Department of Anthropology, Pennsylvania State University, State College, USA; 9 Institute of Geochemistry and Petrology, ETH Zürich, Zürich, Switzerland

**Keywords:** Environmental impact, Geomorphology

## Abstract

Soil sustainability is reflected in a long-term balance between soil production and erosion for a given climate and geology. Here we evaluate soil sustainability in the Andean Altiplano where accelerated erosion has been linked to wetter climate from 4.5 ka and the rise of Neolithic agropastoralism in the millennium that followed. We measure in situ cosmogenic ^14^C directly on cultivated hilltops to quantify late Holocene soil loss, which we compare with background soil production rates determined from cosmogenic ^26^Al and ^10^Be. Our Monte Carlo-based inversion method identifies two scenarios to account for our data: an increase in erosion rate by 1–2 orders of magnitude between ~2.6 and 1.1 ka, or a discrete event stripping ~1–2 m of soil between ~1.9 and 1.1 ka. Coupled environmental and cultural factors in the Late Holocene signaled the onset of the pervasive human imprint in the Andean Altiplano seen today.

## Abstract (web summary)

The assessment of soil sustainability in prehistoric times requires comparing millennium-scale erosion rates with geological background rates. Here, the authors apply in situ cosmogenic ^14^C, ^10^Be, and ^26^Al to reveal rapid soil erosion on the Andean Altiplano in response to Late Holocene climate change and the onset of agropastoralism.

## Introduction

Hillslopes co-evolve with landscapes and vegetation communities to strike a balance between rates of soil production and soil erosion over geological timescales^1–3^. Soil sustainability is a function of this long-term mass balance that culminates in a steady-state soil thickness for a given hillslope curvature as set by climate and geologic setting^1,2,4^. Changes to climate or land use can accelerate the loss of soil, diminish its biological productivity and compromise sustainability as erosion outpaces soil production. Past changes in surface erosion rate are a direct result of how soils respond to environmental change, but with systematic observations of soil erosion rarely more than a few decades long, the lack of data over timescales relevant to soil formation (i.e., 10^2^–10^4^ yr) has hindered a synoptic understanding of soil evolution where natural and anthropogenic forcing intersect.

Given that long-term soil production and erosion tend towards a balance, a compelling evaluation of soil sustainability is to quantify and compare erosion with geological background rates^5^. To this end, vital gains have been made by extracting sedimentary records from floodplains, lakes and estuaries^6,7^. However, this method relies on a close relationship between sediment source and sink, which can be compromised by sediment-transport filtering effects that bias the stratigraphic record^8^. A general difficuly for studies of soil sustainability lies in discriminating the earliest impacts of humans from climate forcing, albeit much evidence points to the coupling of these factors at least during the last few millennia^9–11^. Climatic variations since the last glacial–interglacial transition were a key driver of the technological and social transformations that spawned farming across multiple centres during the Neolithic^12–14^. This link implies humans were exploiting new capacities in their environment and it underscores connections between climate and the impacts of land use on soil, plants and the atmosphere^9,10,15–18^. Proponents of an ‘Early Anthropocene’ point to episodes of soil depletion during agriculture’s pre-industrial rise^9,10,15–18^. If the relationship between the earliest human-driven coupled with climate-driven impacts is a potential basis for assigning the Early Anthropocene, then assessment of soil sustainability across multiple centres of Neolithic agriculture points a way forward.

The Titicaca Basin on the Andean Altiplano is an early centre of agricultural development and its archaeology and palaeoclimate have long been in the research spotlight^19–26^. Some early studies directly link cultural development to climate and environmental change, suggesting massive landscape modification prior to the collapse of both the Tiwanaku (~1 ka) and Inca (~0.5 ka) civilizations^19,27–29^. Others have emphasized drastic soil erosion during the past century^30^. The debate is sustained in part, because no evaluation of soil sustainability has ever been conducted and the erosional dynamics arising from climate and land use remain unquantified.

**Fig. 1:**
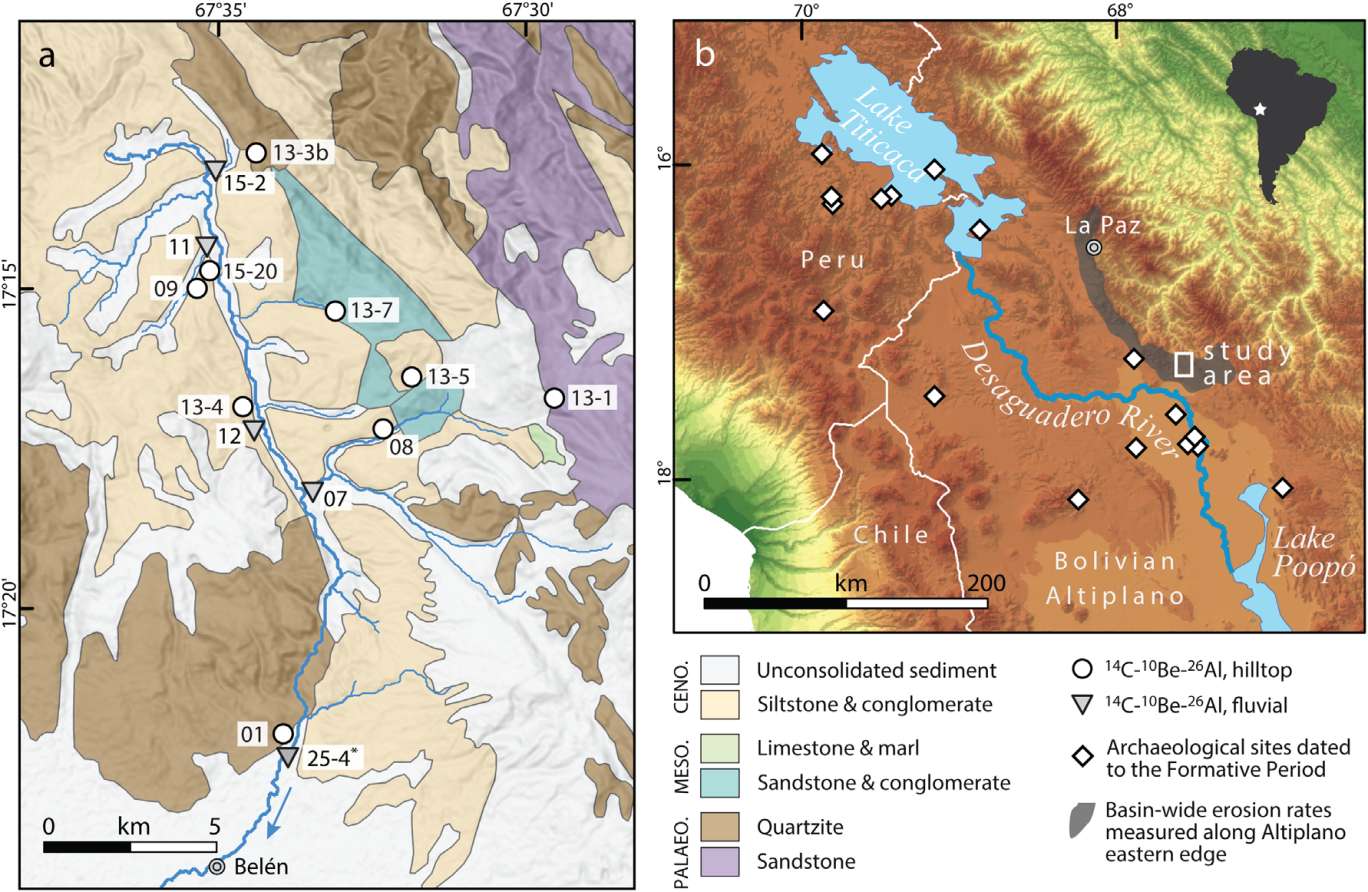
Study area and sampling locations in the southern Titicaca Basin, central Altiplano. **a** The Belén River draining southwards from the Altiplano’s eastern rim and the locations of our samples; sample 25-4 is from Hippe et al.^81^. Rock types are generalized according to their Palaeozoic, Mesozoic or Cenozoic age; geologic map modified after refs. ^82,83^. **b** Southern Titicaca Basin and central Altiplano (South America top-right inset) showing study area (white square) 15–30 km from the Desaguadero valley; 18 regional catchments with basin-wide erosion rates (grey shading, Supplementary Table 6); and 17 archaeological sites (white diamonds) radiocarbon-dated to the start of Formative Period in the Titicaca Basin^44^.

**Fig. 2:**
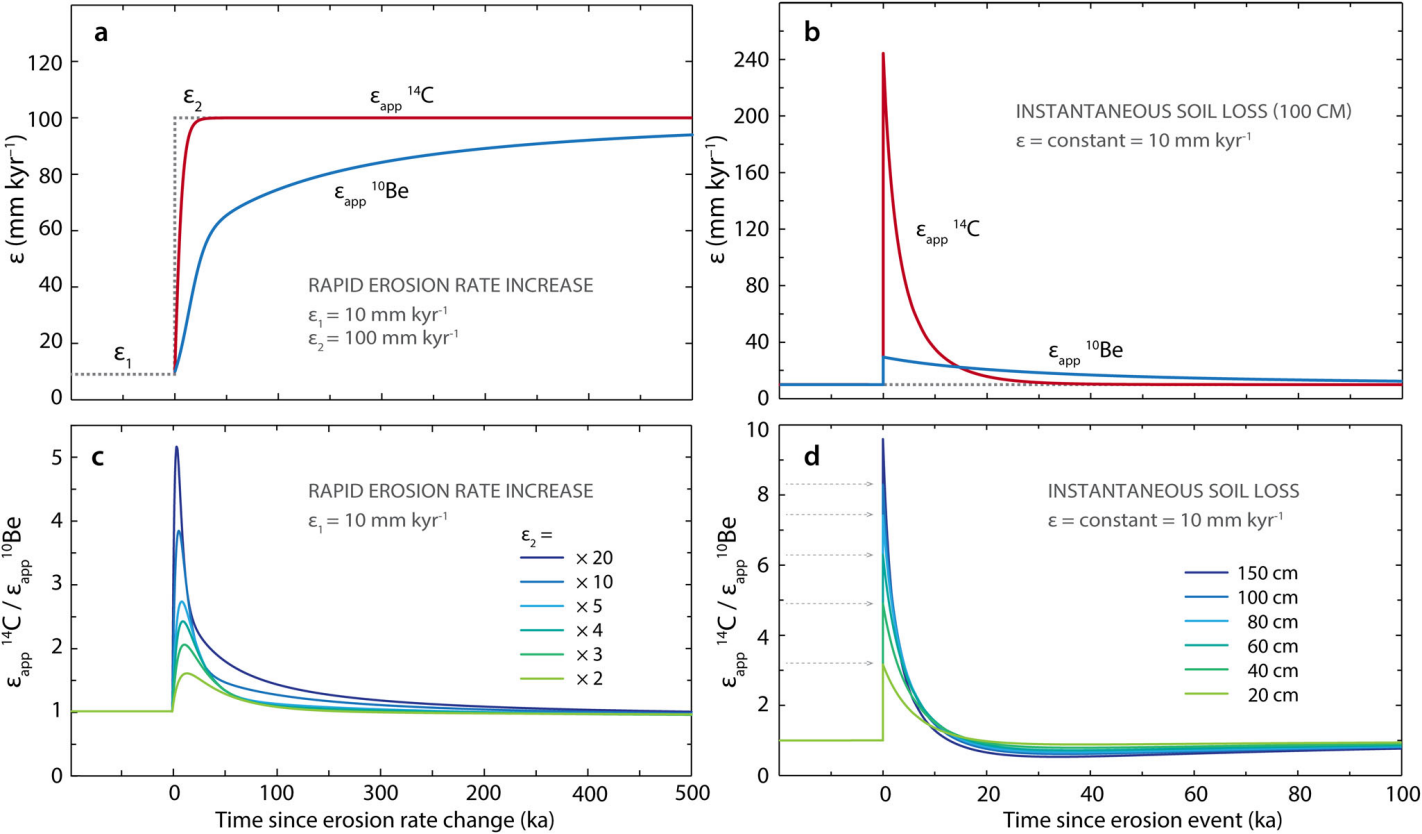
Sensitivity of the ^14^C-^10^Be chronometer for detecting landscape transience. Thanks to their differing half-lives, apparent erosion rates (*ε*_app_) calculated from the measured ^14^C and ^10^Be surface concentrations will deviate in the case of **a** an acceleration in erosion rate (from *ε*_1_ to *ε*_2_), or **b** an instantaneous loss of a soil layer. As ^14^C is more sensitive to the erosional perturbation, the ratio of apparent erosion rates *ε*_app_^14^C/*ε*_app_^10^Be increases immediately after the perturbation (**c**, **d**). Such events are best detected over Holocene timescales and before ^14^C re-establishes isotopic steady state (after ~20 kyr). For better visibility, arrows in **d** mark the maximum of the *ε*_app_^14^C/*ε*_app_^10^Be for each given depth of soil.

Here we evaluate soil sustainability on the Altiplano by measuring cosmogenic radionuclides that accumulate in near-surface rock and soil as a function of exposure to secondary cosmic rays. Nuclides are lost by radioactive decay and surface erosion; the faster a surface erodes, the lower will be its nuclide abundance and the response time to a change in erosion rate (known as erosional transience) is governed primarily by the nuclide half-life and production rate. We measured in situ-produced cosmogenic ^14^C, ^26^Al and ^10^Be in hilltop soil and bedrock and in river sediments of the eastern central Altiplano, south of Lake Titicaca (Fig. 1), and we calculated the apparent erosion rate for each nuclide (i.e., the erosion rate assuming steady erosion; see ‘Methods’). The span of half-lives (5.7 kyr, 0.7 Myr and 1.4 Myr for ^14^C, ^26^Al and ^10^Be, respectively) allows resolving erosion rates over variable timescales employing the long-term ^10^Be-^26^Al chronometer (~10^4^–10^5^ yr) and the short-term ^14^C-^10^Be chronometer (~10^2^–10^3^ yr). If the two chronometers show different apparent erosion rates, we can deduce erosional transience over Holocene (anthropogenic) timescales^31–34^ (Fig. 2). To resolve the timing and magnitude of transience, we use a Markov-chain Monte Carlo (MCMC) inversion model to identify erosion histories that are compatible with the measured nuclide abundances. This in turn enables us to surmise potential causes in light of the known palaeoclimatological and archaeological records.

The Altiplano is an internally drained high plateau stretching ~1000 km along the broadest part of the Andes mountain belt (Fig. 1). Palaeoclimate records show that climate in the late Pleistocene and Holocene fluctuated at both the orbital and millennial timescales^35–37^. A dry Early Holocene culminated with intense aridity at 7.9–4.5 ka^38^ followed by wetter conditions over the past few thousand years^35^ persisting until today. The wetter climate from about 4.5 ka caused a rising Lake Titicaca to overspill into the Desaguadero River at 3.6–3.5 ka^36,39^ (Fig. 1b).

Archaeological research has documented hunter–forager populations in the Andean Altiplano from the terminal Pleistocene and between 10 and 3.5 ka, although population densities remained low largely due to environmental variability^40,41^. Wetter conditions across the Altiplano during the late Holocene was synchronous with a socioeconomic transition from extensive mobile foraging to increasingly sedentary agropastoralism^19,42,43^. This Neolithic (Formative Period) transition spanned 3750 and 2940 cal. yr BP according to a Bayesian analysis of ^14^C ages from archaeological contexts^44^. The adoption of food production during this time of environmental change led to a significantly modified Altiplano landscape. Land clearing, tilling and irrigation for agriculture and pastoralism rapidly took hold, especially around Lake Titicaca, where consistent population growth prompted the development of increasingly large and intensive farming infrastructure including raised fields, irrigation channels, terraces and reservoirs^42,45^.

We focus on the Belén River catchment (~17°16ʹ S, ~67°34ʹ W), part of the Desaguadero catchment that straddles the eastern rim of the Altiplano ~150 km southeast of Lake Titicaca (Fig. 1a, b). Elevations span ~3800–4500 m and the relatively subdued relief reflects variations in erodibility among the Palaeozoic to Cenozoic rocks, yielding smoothly rounded hillslopes with thin stony soils developed on weaker bedrock interspersed with outcrops of resistant sandstone and quartzite up to ~1 m high. The sparse, semi-arid vegetation cover is characterized by *Distichia* tussock grasses and *Polylepis* and *Buddleja* shrub remnants^21^. Abundant traces of past and contemporary traditional agropastoral activity in the area^21,46^ are seen in extensive stone structures including livestock enclosures and low walls marking field boundaries (Supplementary Fig. 1). However, the origin and age of these structures remains largely unknown^46^. The study area is located ~15–30 km from the Desaguadero river valley (Fig. 1b) and is crossed by a major eastward route from the Altiplano to the La Paz River and Amazonia beyond. Based on several archaeological studies conducted within 50 km^47–49^, we know the region was inhabited by foragers from 10 ka onwards and by agropastoralist communities starting ~3.4 ka ago.

## Results

### Soil erosion rates on the 10^3^ to 10^5^ yr timescale

We sampled hilltop bedrock (*n* = 3) and soil (*n* = 6) to constrain point rates of surface lowering. The stony soils comprise rounded cobbles in a sandy matrix and, in places, platy fragments of siliceous hardpan overlying Cenozoic siltstone (Supplementary Fig. 2). In four small tributaries draining the sampled hilltops, we also collected river sand (*n* = 4, Fig. 1). All samples were analysed for cosmogenic ^10^Be, ^26^Al and ^14^C in their quartz fraction (Supplementary Tables 1 and 2) and the concentrations of each nuclide were converted to a corresponding apparent erosion rate (see ‘Methods’).

**Fig. 3:**
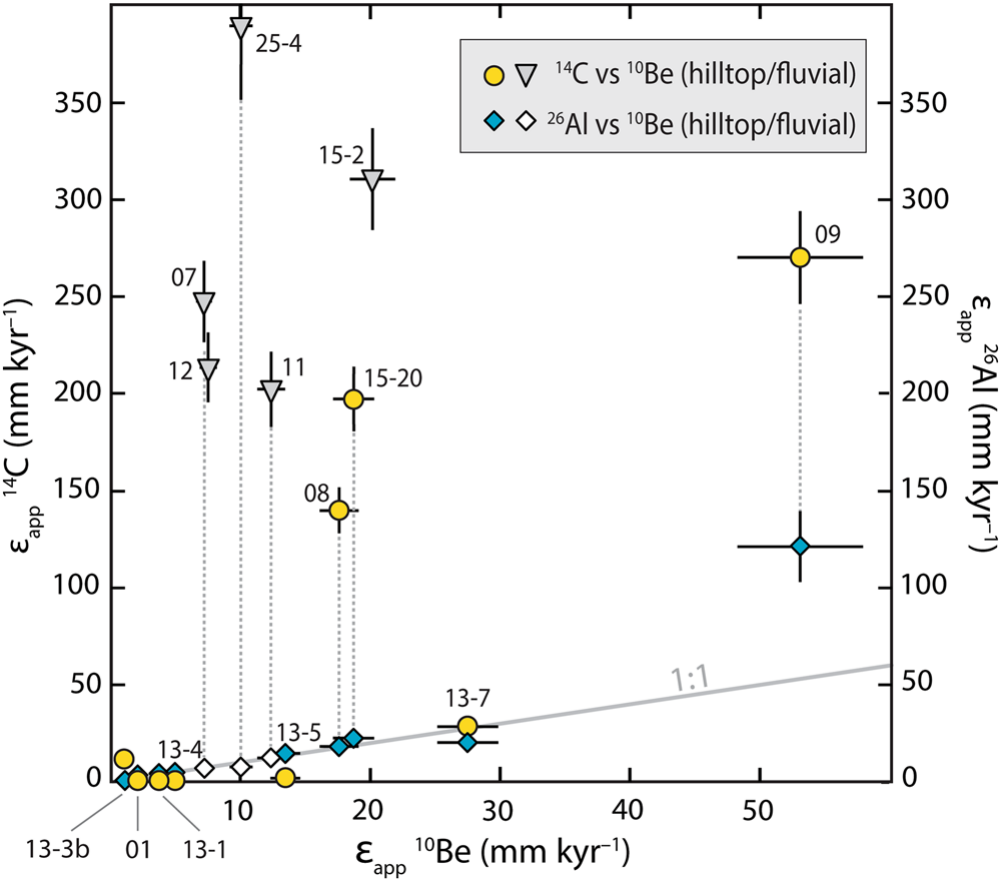
Apparent erosion rates (*ε*_app_) determined from the measured ^14^C, ^26^Al and ^10^Be concentrations. Sample pairs offset from the 1 : 1 grey line (i.e., yellow circles and grey triangles) indicate disequilibrium due to higher ^14^C apparent erosion rate relative to ^10^Be—a signal we attribute to erosional transience. The ^26^Al apparent erosion rate of sample 09 is considered an outlier (see ‘Methods’). Error bars indicate ±1*σ*.

Apparent erosion rates obtained for the long-lived nuclides (^10^Be and ^26^Al) are in good agreement (Fig. 3 and Supplementary Table 3), except for sample 09, which yielded an anomalously low ^26^Al concentration (see ‘Methods’). The ^10^Be-^26^Al apparent rates of long-term hilltop lowering vary according to bedrock erodibility with poorly lithified rocks of Cenozoic and Mesozoic age eroding faster (17.6 ± 1.5 to 53.1 ± 4.8 mm kyr^−1^; samples 08, 09, 13-7, 15-20) than the stronger Palaeozoic rocks (<3.7 mm kyr^−1^; samples 01, 13-1). Palaeozoic clasts recycled into the younger conglomerates also fall among the slowest erosion rates (0.7 ± 0.1 to 14.7 ± 1.3 mm kyr^−1^; samples 13-3b, 13-4, 13-5). The ^10^Be and ^26^Al apparent erosion rates obtained from fluvial sediment samples span a range similar to the point rates (7.2 ± 0.6 to 20.2 ± 1.7 mm kyr^−1^).

The short-term apparent erosion rates obtained from ^14^C for the three bedrock samples (01, 13-1, 13-7) are overall concordant with the ^10^Be-^26^Al rates (Fig. 3), indicating steady erosion throughout the Holocene. For the stony soil samples, ^14^C-derived apparent erosion rates measured in the conglomerate clasts (samples 13-3b, 13-4, 13-5) also agree with the ^10^Be-^26^Al rates. In contrast, the three other hilltop soil samples (08, 09, 15-20, comprising sandy matrix and siliceous hardpan chips weathering out from the weak siltstones) plus all four fluvial samples reveal a pronounced offset between apparent erosion rates for ^14^C and those for the longer-lived ^26^Al and ^10^Be. The ^14^C apparent erosion rates of 140 ± 2 to 270 ± 23 mm kyr^−1^ (hilltop samples) and 202 ± 17 to 311 ± 26 mm kyr^−1^ (fluvial samples) are up to an order of magnitude faster than the corresponding ^10^Be erosion rates (Fig. 3). This disparity indicates that a major episode of erosional transience has occurred. Accordingly, no offset occurs in the bedrock, because no soil existed on those surfaces. The absence of any measurable offset in the conglomerate clasts is a function of their higher erosional resistance relative to the weaker matrix cement, which leads to preferential evacuation of the fine-grained matrix material from these hillslopes via rainsplash and sheetwash. We suggest the conglomerate clasts have concentrated at or near the surface of a desert pavement developed on the stony soils^50^, hence accumulated ^14^C for thousands of years longer relative to the surrounding matrix.

### Timing and amplitude of erosional transience

To identify the potential magnitude and timing of the accelerated erosion, we devised two limiting-case scenarios as follows: (1) a simple step change in erosion rate with an instantaneous shift at a specific time and (2) an abrupt erosional spike that rapidly removes soil of variable thickness at a specific time before returning to a constant pre-spike erosion rate. These limiting cases describe how a major landscape perturbation may have affected surface erosion rates. To test these two scenarios and delineate probable erosion histories, we apply a joint MCMC inversion model (Supplementary Fig. 3) to the three hilltop and four fluvial samples with offset ^14^C-^10^Be concentrations assuming a concurrent timing of change for all locations.

**Fig. 4:**
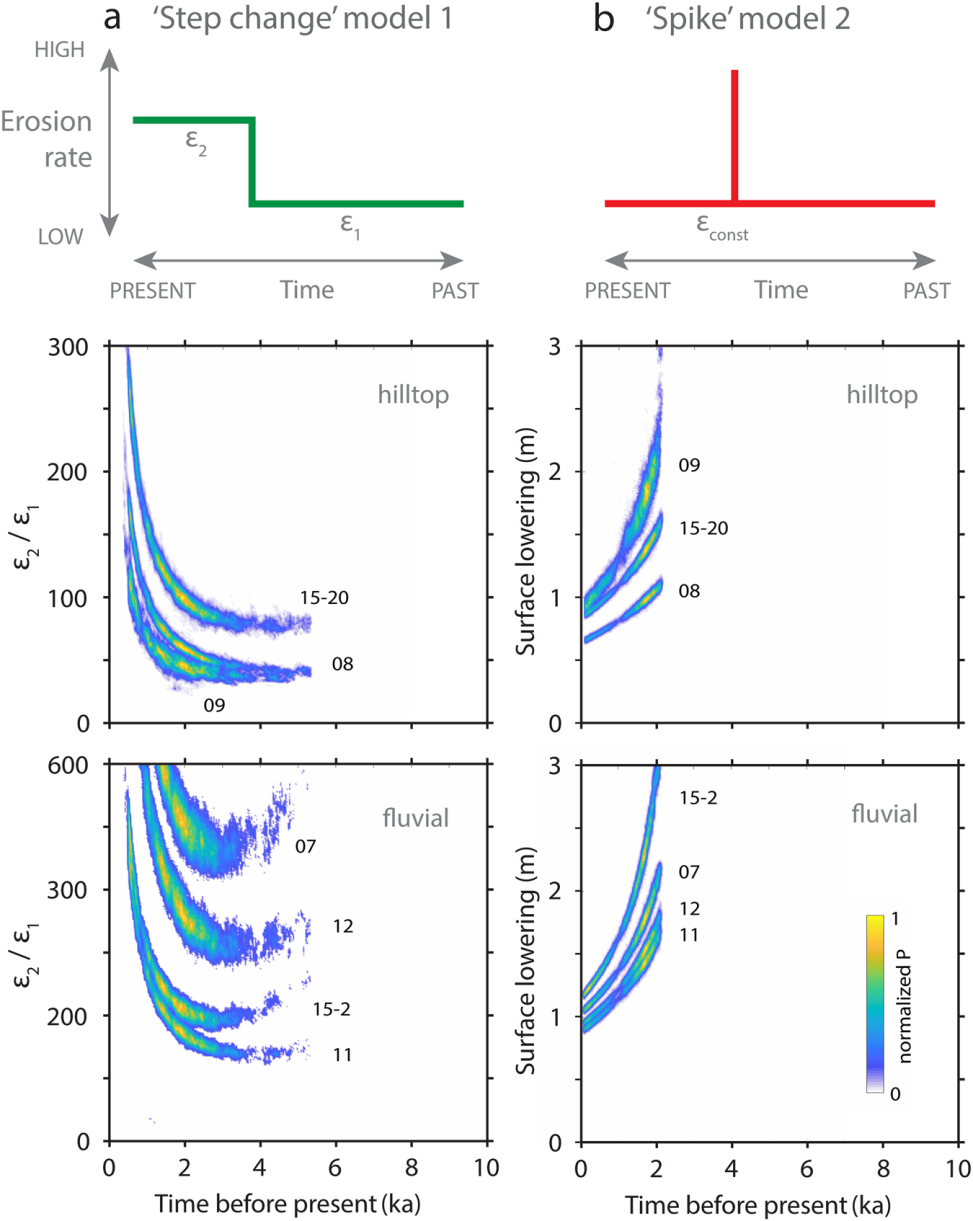
Results of the MCMC inversion modelling of the two limiting-case scenarios. Model results are based on a joint inversion of three hilltop samples and four fluvial samples that show a ^14^C-^10^Be offset. We assume that all samples record the same timing of the perturbation but have variable bedrock-controlled past and present erosion rates. **a** The step model shows the ratio of present to past erosion rates (*ε*_2_/*ε*_1_) over time illustrating the possible timing and amplitude of erosion rate increase in the past. Note the change in scale of the *y* axis in the plot for the fluvial samples, which indicates an overall larger amplitude of perturbation. **b** The spike model shows the possible magnitude of surface lowering (in m) over time. The colour ramp applies to all plots and shows the density of accepted models normalized to the highest value such that the most frequent results are denoted by *P* = 1. Both limiting-case scenarios indicate that a strong landscape perturbation occurred in the Late Holocene.

Results from the ‘step change’ model point to a major acceleration in erosion rates of two orders of magnitude. The amplitude of this acceleration varies with timing, but the most likely models (interquartile range, IQR) show a ~40- to 150-fold increase in hilltop erosion rates between 2.6 and 1.1 ka (hilltop samples only, Fig. 4a and Supplementary Table 4). Results from the ‘spike’ model suggest an erosion pulse that lowered hilltops by ~0.8 to 2.0 m between 1.9 and 1.1 ka, IQR (hilltop samples only, Fig. 4b and Supplementary Table 5). Including the fluvial samples, the models show an up to 730-fold increase in erosion rates or a hilltop lowering of up to 2.5 m (Fig. 4a, b and Supplementary Tables 4 and 5).

Relaxing our model constraints to allow the time of change to vary between sampling sites yields a similar amplitude of change over a slightly wider time interval (Supplementary Fig. 4 and Supplementary Tables 4 and 5). A hybrid mix of the two limiting cases is also compatible with our results, although pronounced acceleration in erosion is common to all modelled scenarios. Alternatively, oscillating erosion rates driven by cyclical climate can also be envisioned, yet this would still require an exceptionally strong Late Holocene landscape perturbation to explain the offsets in our ^14^C-^10^Be data. Either way, we conclude that Late Holocene erosion rates were one to two orders of magnitude faster compared to erosion during the preceding tens of thousands of years.

Apart from erosional transience, other mechanisms have the potential to deplete ^14^C relative to ^10^Be, such as intense vertical mixing of hilltop soils or intermittent burial. However, by modelling a range of depth-dependent mixing intensities, we can exclude mixing as the source of the measured ^14^C depletion (Supplementary Fig. 5). Similarly, temporary burial of the fluvial sediment during transport can be excluded as a cause of the ^14^C offset, because the floodplains are too young and too thin to provide for significant ^14^C decay^51^. Notwithstanding that gullying potentially contributes deeply buried particles with low ^14^C, the consistency of the ^14^C-^10^Be offset (45–65 %) in both our hilltop soils and fluvial sediments suggests that a shared landscape-wide signal of transience has been transmitted from the hillslopes to the river system.

## Discussion

A key advantage of our multi-nuclide approach to quantifying soil erosion is that measurements are conducted directly at the sites of soil production. This makes it possible to evaluate accurately the balance between production and erosion without the need of an assumed coupling between sediment source and depositional sink—an assumption that is central to assessments of soil sustainability based on records retrieved from floodplains, lakes or estuaries^6,7^ and references therein. When dealing with sedimentary archives, temporary sediment storage or even the dynamics of sediment transport itself can interrupt and distort the erosional and depositional record of events leading to a highly filtered stratigraphic record^8,52,53^.

**Fig. 5:**
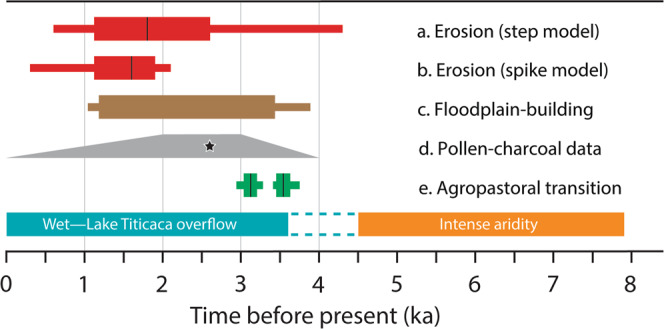
Altiplano history relating to climate, land use and deposition/erosion. Timeline showing (**a**, **b**) ‘step change’ model and ‘spike’ model outputs as box-whisker plots (IQR and 5–95 percentile range, median black line). **c** Range of floodplain radiocarbon ages as box-whisker plot (±1 and ±2*σ* error), indicating increased sediment supply to valley floors via hillslope erosion. **d** Pollen and charcoal data^63^, indicating onset of human-induced vegetation change from 4 ka, peak abundance of food-crop pollen (Chenopodiaceae/Amaranthaceae) and charcoal at ~2.6 ka (star) followed by decline in charcoal (despite high lake levels), implying comprehensive deforestation from ~2 ka. **e** Timing of the agropastoral transition start and end^44^ as box-whisker plots (±1 and ±2*σ* error, and median black line). At base, schematic climate scenario describing mid-Holocene aridity (7.9–4.5 ka)^36,39^ and succeeding wetter conditions that raised lake levels (~4.5–3.6 ka) in the Titicaca Basin.

The steady-state background rate of soil production and erosion provides a benchmark against which we can evaluate soil sustainability in the southern Titicaca Basin. For our purposes, this benchmark is set by the basin-wide ^10^Be-derived erosion rate of 10.8 ± 7.6 mm kyr^−1^ (±1*σ*) averaged across 18 catchments including the study area (10.1 ± 0.9 mm kyr^−1^, sample 25-4) (Fig. 1a and Supplementary Table 6). Together, these data integrate the past ~60 kyr and so represent erosion rates of the late Pleistocene–Holocene, as controlled by climate and lithology. The Late Holocene surge in erosion rate was, by comparison, an order of magnitude faster and evident at the scale of the catchment, subcatchment and hilltops (Fig. 3). Soils stripped from hillslopes were transported via rainsplash and sheetwash into temporary storages at the base of slopes and in floodplains, and these accumulations exist along the Belén valley today. Six conventional radiocarbon dates^51^ spanning 3440–2540 cal. yr BP (2*σ*) or 2110–1180 cal. yr BP (2*σ*) indicate that the silty floodplain deposits (up to 4 m thick) are direct counterparts to the accelerated erosion of the hillslope soils (Fig. 5). The floodplains are currently incised presumably due to the decline in sediment supply rate from hillslopes^51^. This fall in sediment supply is a scenario described by our spike model (Fig. 4).

Investigations of the palaeoclimate and archaeology of the Titicaca Basin show the Late Holocene to be a time of sweeping transformation on two counts: (1) a wetter climate from the mid-Holocene with the overflow of Lake Titicaca and watering of the Desaguadero River valley and (2) the transition from hunter–forager subsistence to agropastoralism in the Formative (Neolithic) Period at ~3.5 ka (Fig. 5). The rising water level in Lake Titicaca after 4.5 ka confirms that runoff was increasing due to wetter conditions, at least in the northern Altiplano^36,39,54^. From an analysis of palaeohydrological proxies along a north–south transect, Abbott et al.^55^ suggest that wetter conditions came to the southern Titicaca basin ~2 kyr later (~2.5 ka) via a progressive southward shift in wet-season convection. This points to a convergence in the timing of climatic amelioration and flooding along the Desaguadero valley over the period ~3.5 to 2.5 ka. In response, the sparse plant cover that developed under aridity would have become denser, thereby reducing erosion in the uplands^56–58^. The onset of soil thinning during the interval ~2.6 to 1.9 ka followed this chain of events, although the temporal uncertainty allows for a lag of up to several centuries after the upswing in effective moisture (Fig. 5). We cannot constrain further the role of climate with our results, yet it seems unlikely to have been the sole trigger for soil thinning given the huge magnitude of soil loss indicated by our data and the emergent anthropogenic influences across the Titicaca Basin at this time.

Archaeological research in the central Altiplano demonstrates the adoption and development of camelid pastoralism supplemented by quinoa and tuber cultivation from ~3.4 ka onwards^47,49^. Initially characterized by dispersed domestic and funerary mounds formed by overlapping pastoralist residential bases, this was eventually replaced by dispersed homesteads. Human settlements tied pastures to dryland farming communities, structuring a traditional subsistence system that has survived to modern times. We view the onset of agropastoralism in this region as essentially coeval with the reported sedimentary record and compatible with a model of synchronous cultural and environmental change that has been previously applied to Neolithic societies^59,60^. If it is the case that agropastoralism developed first in the highlands west of Lake Titicaca before spreading into the southern Titicaca Basin^44^, then the capacity for upland agriculture (in our study area) already existed at the time of the demographic expansion down the Desaguadero valley. The best estimate of the agropastoral transition, 3.75–2.94 ka^44^, implies that soil-thinning was preceded by several centuries to a millennium or so. This delay (if not a function of imprecise dating) possibly reflected the demographic response time to a boost in resources^61,62^, or perhaps an initial phase of sustainable farming prior to the onset of practices that led to irreversible soil loss on the uplands.

It is well established that soils become vulnerable to rainsplash and sheetwash erosion when subjected to fire, grazing or cultivation, which disrupt the capacity of plants to bind the soil. Although Andean societies of the last millennium continued to degrade soils^19,27–29^, our data suggest that they inherited a resource that was already severely depleted. Analyses of pollen and charcoal in a drill-core spanning the past 27.5 kyr shows that fire had long been a part of the regional ecology prior to humans^63^. Early foragers probably used fire to convert forest and woodlands to grasslands^64^, but Paduano et al.^63^ argue that burning had no measurable effect on soil erosion until about 2600 cal. yr BP when humans had increased the vulnerability of upland soils by clearing natural vegetation, introducing domesticated camelids, and cultivating hillslopes for food crops such as quinoa^43^. Similarly, we hypothesize that in the Belén catchment, soil thinning was linked to deforestation, land clearance for agriculture and herding intensification in a context of climatic variability^63^.

Although we cannot separate the relative influence of environmental vs. anthropogenic forcing on long-term soil sustainability, we question whether such partitioning is even possible given the technological and social transformation unfolding in the Andes at the time^45^. A more persuasive argument is that Andean agropastoralist communities were responding to climate variability by modifying their environment in highly dynamic ways^65^. The catastrophic soil depletion documented here is therefore a result of the transformative effects on their landscape and is plausibly tied to the onset of the Early Anthropocene in the Andean Altiplano. Alongside the broadscale human-driven transformation of Earth’s surface recognized around 4–3 ka^66,67^, our findings support a growing challenge to the idea of a late Anthropocene tied solely to the industrial era^12^.

## Methods

### Sample collection and preparation

We collected a total of 13 samples including hilltop bedrock (*n* = 3), hilltop stony soil (*n* = 6) and river sand (*n* = 4). All hilltop samples were collected at the crests of hills to ensure sampling of in situ material not subject to downslope transport. Bedrock samples were collected from outcrops of Silurian sandstone (13-1), Devonian quartzite (01) and Cretaceous sandstone (13-7). A maximum sample thickness of 5 cm below the surface was taken. Soil samples comprise amalgamated clasts from Oligocene–Miocene conglomerates (100–150 mm diameter, 13-3b, 13-4, 13-5), matrix sand from Oligocene–Miocene conglomerate (sieved to 0.2–1 mm, 08) and amalgamated siliceous chips from hardpan soils developed on Oligocene–Miocene siltstone (20–60 mm; 09, 15-20). Siliceous hardpan is a pedogenic concentration of opaline silica commonly found in arid landscapes^68^. All rock and clast samples were crushed and sieved to a grain size of <0.6 mm. Quartz was isolated via leaching with hydrochloric acid (HCl) and weak hydrofluoric acid (HF).

### Cosmogenic ^10^Be-^26^Al analysis

About 22–30 g of each clean quartz sample was spiked with ~250–300 μg of ^9^Be carrier and dissolved in concentrated HF. Be and Al were extracted by ion exchange chromatography including separation of Fe in an anion column, isolation of Al in an HCl-based cation column and separation of Be in a second cation column set-up using oxalic acid to remove elements other than Be and HNO_3_ for Be elution. Be and Al were precipitated as hydroxides and transformed into oxides at 1000 °C and 950 °C, respectively. Measurements of the ^10^Be/^9^Be and ^26^Al/^27^Al ratios were performed at the ETH Zürich TANDY accelerator mass spectrometer. Measured ratios were normalized to ETH S2007N standard for Be and ZAL94N for Al^69^. A long-term process blank with a ^10^Be/^9^Be ratio of (3.5 ± 0.3) × 10^−15^ (±1*σ*) was subtracted from the measured ^10^Be/^9^Be ratio. The total Al concentration was determined by inductively coupled plasma quadrupole mass spectrometry measurements at ETH Zürich on sample aliquots taken immediately after quartz dissolution. All results are summarized in the Supplementary Table 1.

### Cosmogenic ^14^C analysis

In situ ^14^C was extracted from aliquots (∼3–5 g) of the same purified quartz samples analysed for ^10^Be and ^26^Al. Samples 01, 07, 08, 09, 11 and 12 were analysed in 2012 at the ETH Zürich extraction system as outlined in ref. ^70^. Samples 13-1, 13-4, 13-5, 13-7, 15-2 and 15-20 were analysed in 2018 on a new extraction system at ETH Zürich^71^. In both systems, atmospheric ^14^C adsorbed to crystal surfaces is removed by sample preheating (~500 °C) followed by release of cosmogenic ^14^C from the quartz crystal lattice by high-temperature diffusion (~1600–1670 °C for 2–3 h). The CO_2_ samples were measured without prior graphitization at ETH Zürich MICADAS accelerator mass spectrometer system equipped with a gas ion source^72^. Data reduction followed the protocol of ref. ^73^ including the subtraction of a long-term process blank applicable to the time of extraction (Supplementary Table 2). The reason for a 17% excess in ^14^C for bedrock sample 01 is unknown. However, two independent ^14^C analyses on separate aliquots of sample 01 yielded identical results within 3% validating the high ^14^C concentration.

### Apparent erosion rates

We convert the measured concentrations of all three nuclides into apparent erosion rates providing point-specific information for the hilltop samples and sub-basin averaged erosion rates for the fluvial samples (Supplementary Table 3). We emphasize ‘apparent’, because this approach yields erosion rates that assume steady-state conditions and negligible loss of nuclides by radioactive decay during the time periods considered here (i.e., the Holocene). Although the latter condition is fulfilled for the slowly decaying ^10^Be and ^26^Al nuclides, the shorter-lived ^14^C might experience some nuclide loss via decay for surfaces eroding as slowly as a few mm kyr^−^^1^. Nevertheless, potential loss of ^14^C through decay will reduce the concentrations measured here by a few percent only and does not affect the results and interpretation of the ^14^C data. Local nuclide production via spallation was determined with the Matlab code of the online calculator v. 2.3^74^ using constant *St* scaling^75^ and sea-level high latitude (SLHL) production rates of 4.01 ± 0.33 at g^−1^ yr^−1^ for ^10^Be, 27.90 ± 2.80 at g^−1^ yr^−1^ for ^26^Al and 12.20 ± 0.89 at g^−1^ yr^−1^ for ^14^C^76^. Muonic production rates at the surface are based on muon interaction cross-sections for ^10^Be-^26^Al^76^ and ^14^C^77,78^. Depth-dependence of nuclide production was modelled via simple exponential functions with attenuation lengths 160, 1500 and 4320 g cm^−2^ for production via spallation, negative muon capture and fast muons, respectively. We assume a density of 2.65 g cm^−3^ for bedrock samples; for all other samples (clasts and river sand), we assume a density of 2.0 g cm^−3^. This is a valid approach also for the clasts, which have higher density but have been exhumed via removal of the lower-density matrix. For sample 09, we determined a ^26^Al concentration that yields a ^26^Al/^10^Be ratio of 3.1, which we consider to be implausibly low. This sample contains significantly lower total Al (∼7 p.p.m.) relative to other samples and 2.4-fold lower total Al than sample 15-20, which was collected from the same lithology at a nearby hilltop (Supplementary Table 1). Consequently, we exclude the ^26^Al for sample 09 from our analysis but this decision has no bearing on our overall conclusions.

### MCMC inversion modelling

We apply an MCMC-based inversion model^34,79^ to investigate the ^14^C-^10^Be disequilibrium observed in seven of our samples and to test the hypothesis that erosional transience may be responsible for the disequilibrium. We employ a Metropolis-Hastings type MCMC model to map parameters that provide the best, weighted least-squares fit to the measured nuclide data (all nuclide systematics are consistent with those used for the erosion rate calculations). The model parameters are [*ε*_1_, *ε*_2/_*ε*_1_, *t*] for the step model and [*ε*_const_, *x*, *t*] for the spike model where *t* is the time of erosion rate change or the erosion event (yr BP), respectively, and *x* is the depth of surface lowering (m). For each model run, 32 random walkers search the parameter space defined as *ε*_1_: 0.1–25 mm kyr^−1^, *ε*_2/_ *ε*_1_: 0.1–1000, *ε*_const_: 0.05–30 mm kyr^−1^, *x* = 0–3 m and *t*: 1–10,000 yr. For the joint inversion, the time *t* is shared by all sample localities, but the erosion rates and sediment loss are allowed to vary from place to place. Each walker begins with a burn-in phase of 5000 iterations that allow for a coarse initial search of the model space. Using the results of the burn-in phase, a detailed search of the model parameters is performed with 40,000 iterations yielding 1.28 million iterations in total (32 × 40,000). Walkers aim for a 40% acceptance ratio. In addition to the joint inversion, we apply individual inversions that allow the time of change to vary between sampling localities (Supplementary Fig. 4). We report the minimum, 0.05, 0.25, 0.50, 0.75, 0.95 and maximum percentiles from 1.28 million accepted iterations for each sample (Supplementary Tables 4 and 5).

### Vertical mixing in soil profiles

Unconsolidated sediments or soils can be affected by mixing processes due to bioturbation and/or cryoturbation. To assess whether the measured depletion of ^14^C in the hilltop samples might result from soil mixing, we employ a numerical model^80^ to investigate changes in ^14^C and ^10^Be concentrations within a variably mixed soil layer. Assuming that mixing diffusivity decreases exponentially with depth, diffusivity at depth *z* may be expressed as:

1 $$m(z)={m}_0\cdot{e}^{-\frac{z}{D}}$$

where *m*_0_ is the diffusivity at the surface and *D* the diffusivity decay depth. We consider that depth-dependent soil diffusivity better represents natural soil-mixing processes than the end-member case of steady erosion and complete uniform mixing^68^. To compare the modelled steady-state depth profiles for ^14^C and ^10^Be in a non-uniform mixed soil layer (Supplementary Fig. 5a, b), we apply a constant erosion rate of 10 mm kyr^−1^ according to the average ^10^Be-derived erosion rate from our study area, a surface diffusivity of 0.1 cm^2^ yr^−1^ and *D* values of 0, 20, 40, 60, 80, 100 and 120 cm. To accommodate the slight variation in nuclide production rates at the different sampling locations, we apply SLHL reference production rates for ^10^Be and ^14^C as given above and scale the measured concentrations accordingly. The deviation of the ^14^C concentration and the ^10^Be/^14^C ratio in a mixed soil layer from a steadily eroding non-mixed surface is calculated for surface erosion rates between 0.1 and 1000 mm kyr^−1^ using the same diffusivity parameters and production rates (Supplementary Fig. 5c).

## Supplementary information

Supplementary Information

## Data Availability

All data are available in the main text or the Supplementary Information.

## Code availability

The Matlab codes we used to perform all model calculations can be accessed from D.L.E. (david@geo.au.dk).
