# Supplementary material for: Cosmogenic in situ 14C-10Be reveals abrupt Late Holocene soil loss in the Andean Altiplano
Source: Nat Commun. 2021 May 5;12:2546. doi: 10.1038/s41467-021-22825-6 (PMC8099901; doi:10.1038/s41467-021-22825-6)
Supplement: Supplementary file 1 — Supplementary Information [file 41467_2021_22825_MOESM1_ESM.pdf]

Supplementary Information

Cosmogenic *in situ* ^{14}C-^{10}Be reveals abrupt Late Holocene soil loss in the Andean Altiplano

K. Hippe, J.D. Jansen, D.S. Skov, M. Lupker, S. Ivy-Ochs, F. Kober, G. Zeilinger, J.M. Capriles, M. Christl, C. Maden, C. Vockenhuber, D.L. Egholm

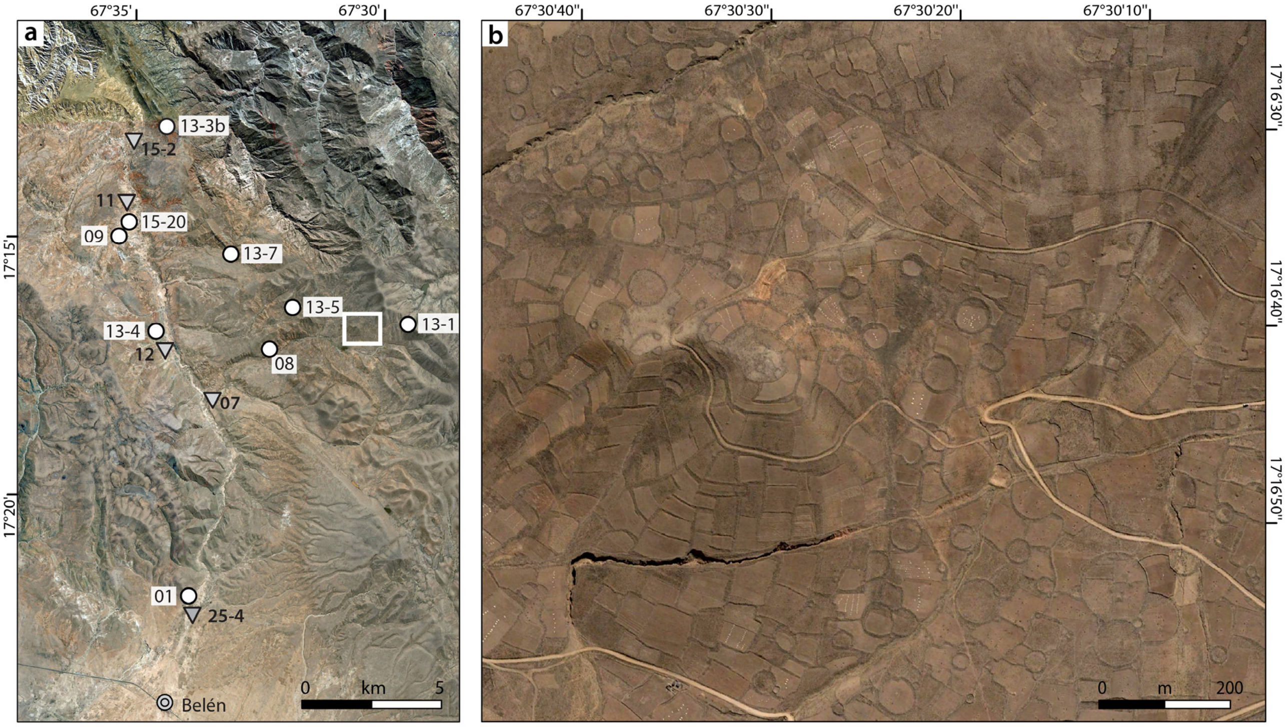

Fig. S1 Traces of agropastoral activity in the study area.

a) Belén catchment with sampling locations; the image section is equivalent to Fig. 1. The white square marks the extent of the enlarged view shown in the right panel. b) Extensive stone structures that can be observed throughout the entire Belén catchment. Structures include livestock enclosures and low walls marking field boundaries; many are still maintained by local communities. The origin of these structures is undated, however, the oldest ones most likely originate from the Late Intermediate Period (i.e., within the last 900 years).

Image source: Google Earth, Map data: Google, Maxar Technologies.

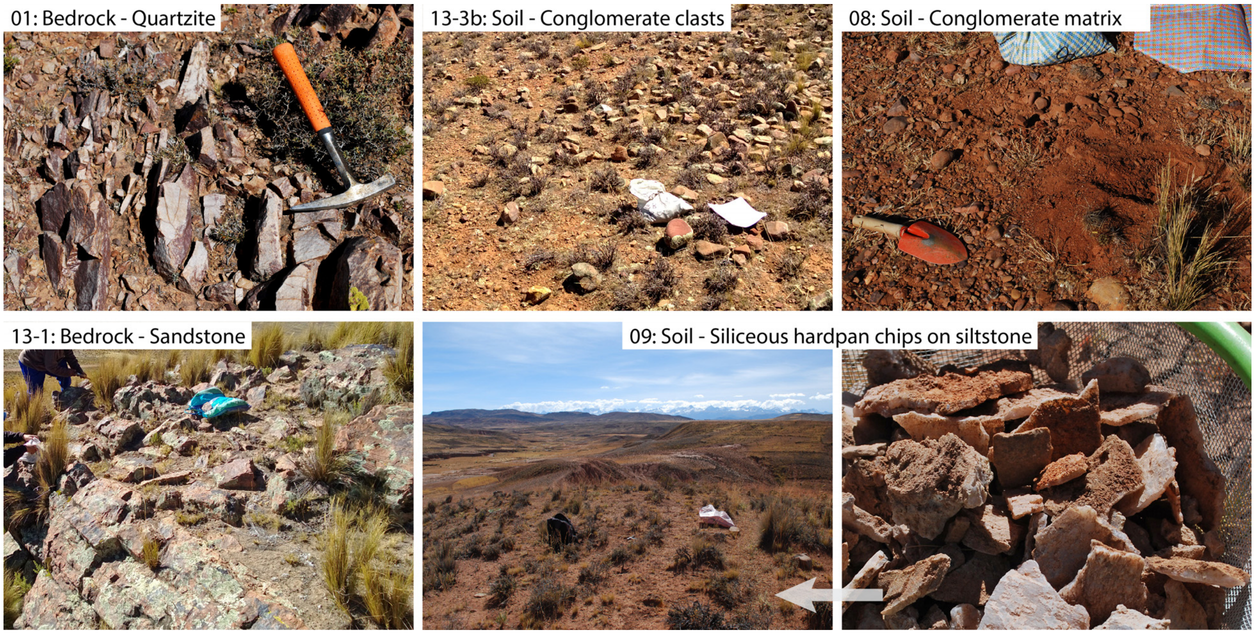

Fig. S2 Representative pictures of collected hilltop bedrock and soil samples. Photos are given with the sample ID and sample type. 01: Near-vertical outcropping Devonian quartzite. 13-3b: Poorly-developed stony soils formed from Oligocene-Miocene conglomerates with accumulation of conglomerate clast on hilltops; sampled clast sizes were on average 100–150 mm. 08: Conglomerate matrix sand collected from Oligocene-Miocene conglomerates; loose sand for sampling was found only in the uppermost 2-3 cm. 13-1: Slightly tilted beds of Silurian sandstone standing out ~1.5 m above the ground. 09: Siliceous hardpan chips (average size 20–60 mm) collected from rounded hilltops of Oligocene-Miocene siltstone.

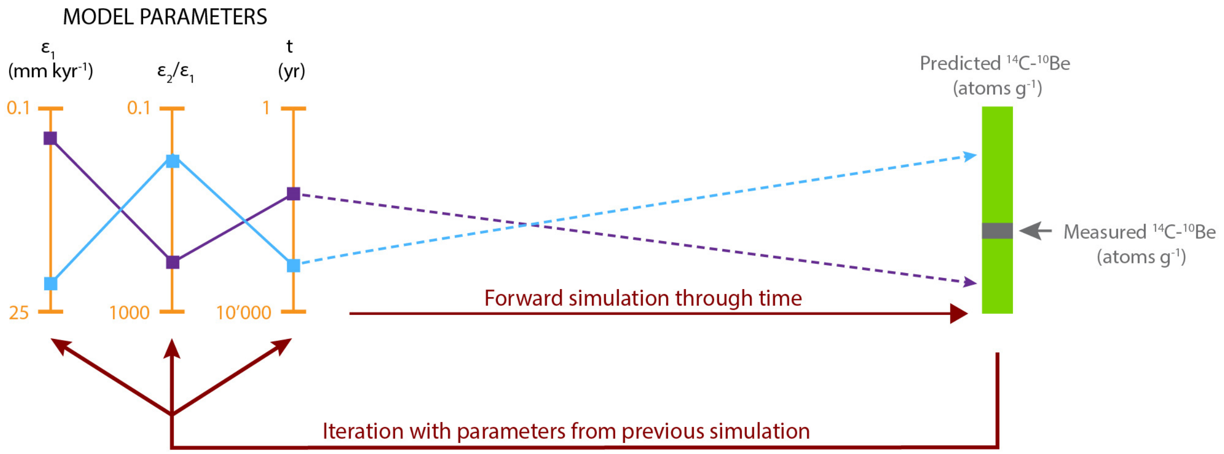

Fig. S3 Concept of the Markov-chain Monte Carlo (MCMC) inversion model[1,2].
The model searches the model space defined by the boundaries of the parameters ε_1, $\varepsilon_2/\varepsilon_1$, and t (for the 'step change' model as shown here) or ε_{const}, x and t (for the 'spike model', not shown here) for the best fit to the measured ^{14}C-^{10}Be nuclide data.

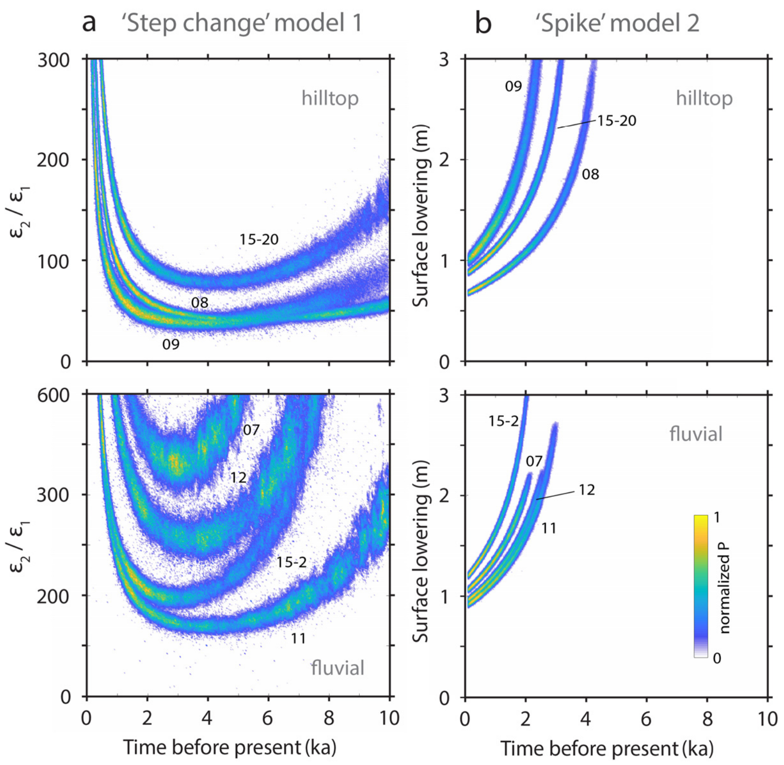

Fig. S4 Results of the MCMC inversion modelling of the two limiting-case using individual inversions that allow for all parameters to vary for each sample.

a) The 'step change' model plots show the ratio of present to past erosion rates ($\varepsilon_2/\varepsilon_1$) over time illustrating the possible timing and amplitude of erosion rate increase in the past. Note the change in scale of the y-axis in the plot showing the fluvial samples. b) The 'spike' model plots show the possible magnitude of surface lowering (in m) over time. The color ramp applies to all plots and shows the density of accepted models normalized to the highest value, such that the most frequent results get P = 1. Both limiting-case scenarios for all samples suggest a very strong landscape perturbation occurred in the late Holocene. Model results for the fluvial and hilltop samples are in good agreement and indicate that the offset cosmogenic nuclide signal was transported from the hillslopes into the channels. However, fluvial samples indicate overall larger amplitude of perturbation.

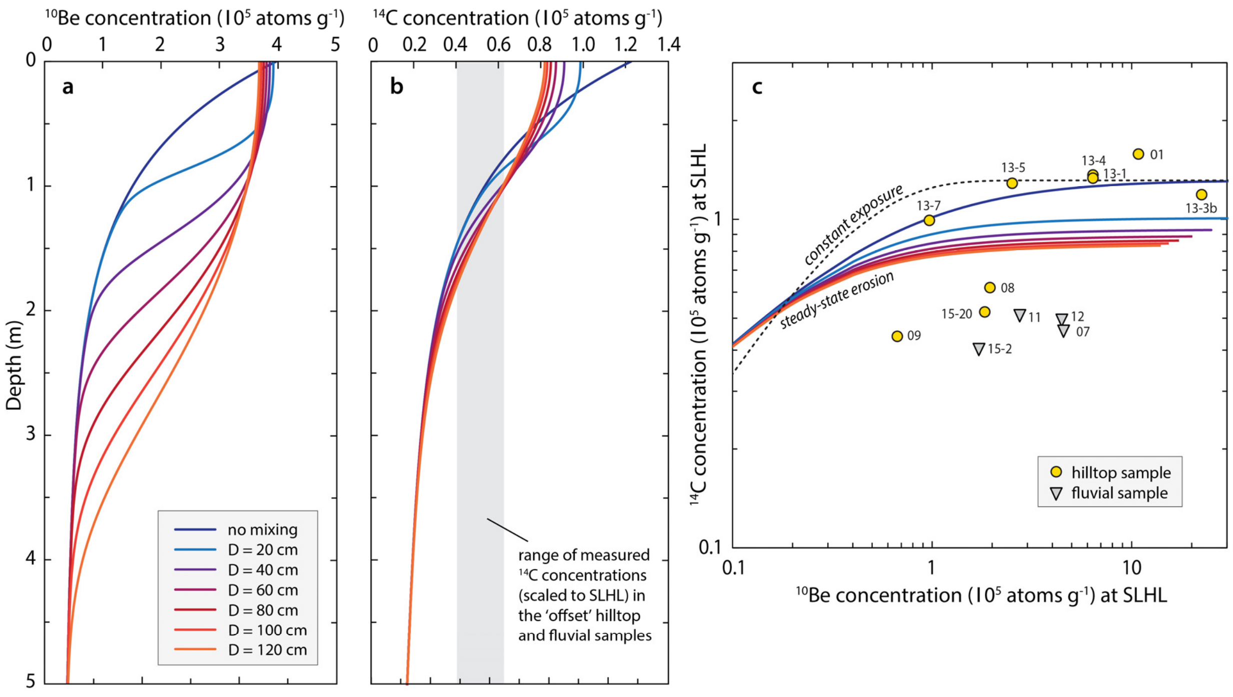

Fig. S5 Surface concentrations and depth distribution throughout a mixed soil layer modelled for ^{10}Be and ^{14}C assuming depth-dependent soil diffusivity[3].

a) ^{10}Be concentrations at the surface are primarily controlled by the surface erosion rate (here 10 mm kyr^{-1}) and remain near the non-mixed surface value even for increasing mixing intensities. At depth, ^{10}Be concentrations reflect a general increase in mixing depth with increasing mixing diffusivities. b) ^{14}C concentrations at the surface decrease with increasing mixing diffusivities and converge towards the non-mixed depth profile already at shallow depths (< 1 m). The latter trend reflects the time component inherent in the diffusivity term, which limits the impact of deep, time-intensive diffusion processes and balances ^{14}C decrease from soil mixing and ^{14}C loss from fast decay. c) ^{10}Be vs. ^{14}C concentration diagram illustrating the lowering of the ^{14}C surface concentrations of a steadily eroding surface with increasing soil mixing intensities (i.e. D-values as shown in a). Samples from non-eroding surfaces should plot along the line of constant exposure. Results show that vertical soil mixing seems insufficient to cause the measured ^{14}C depletion in the hilltop (samples 08, 09, 15-20) and fluvial samples.

Table S1 Sample information and ^{10}Be-^{26}Al data.

Sample ID	Sample type	Lat (°)	Lon (°)	Altitude (m asl)[a]	Sample thickness (cm)	Sample mass (g_{qtz})	^{9}Be carrier (mg)	Measured ^{10}Be/^{9}Be (10^{-12})[b]	^{10}Be/^{9}Be error (%)	^{10}Be conc. (10^{6} at/g)[c]	Total Al (ppm)	Measured ^{26}Al/^{27}Al (10^{-12})[b]	^{26}Al/^{27}Al error (%)	^{26}Al conc. (10^{6} at/g)[c]	$\frac{^{26}Al}{^{10}Be}$
Hilltop bedrock (*) & soil samples															
01*	Quartzite	-17.3660	-67.5635	3880	3	22.10	0.3059	8.54	1.5	7.81 ± 0.12	292	8.08	2.1	52.67 ± 1.25	6.74
08	Congl. matrix	-17.2859	-67.5378	4029	1	25.25	0.3050	1.95	2.2	1.56 ± 0.03	130	3.57	3.0	10.32 ± 0.33	6.64
09	Siliceous chips	-17.2488	-67.5888	3988	1	23.05	0.3059	0.60	3.9	0.52 ± 0.02	7	11.07	10.0	1.64 ± 0.21	3.13
13-1*	Sandstone	-17.2771	-67.4913	4326	2	24.46	0.2528	8.45	1.5	5.77 ± 0.09	334	5.61	1.1	41.78 ± 1.34	7.24
13-3b	Congl. clasts	-17.2141	-67.5728	4112	2	26.10	0.2543	28.44	1.4	18.33 ± 0.25	410	14.14	0.9	129.32 ± 5.51	7.06
13-4	Congl. clasts	-17.2801	-67.5760	4016	3	25.01	0.2525	7.42	1.5	12.18 ± 0.17	387	4.81	1.5	41.55 ± 1.53	8.38
13-5	Congl. clasts	-17.2721	-67.5302	4220	3	24.27	0.2525	3.12	2.0	18.33 ± 0.25	175	3.44	1.6	13.43 ± 0.46	6.26
13-7*	Conglomerate	-17.2561	-67.5506	4051	5	25.18	0.2517	1.14	2.0	4.96 ± 0.07	248	1.26	2.7	6.97 ± 0.28	9.30
15-20	Siliceous chips	-17.2448	-67.5861	3994	1	24.85	0.2517	2.16	2.0	1.45 ± 0.03	17	21.87	2.2	8.39 ± 0.43	5.81
Fluvial samples															
07	Sand	-17.3033	-67.5580	4144	-	29.79	0.3057	5.61	1.8	3.80 ± 0.07	178	4.86	1.4	19.26 ± 0.63	5.06
11	Sand	-17.2359	-67.5829	4061	-	22.19	0.2400	3.09	2.2	2.21 ± 0.05	155	3.52	1.7	12.20 ± 0.42	5.52
12	Sand	-17.2843	-67.5718	4032	-	27.94	0.3396	4.39	3.4	3.53 ± 0.12	-	-	-	-	-
15-2	Sand	-17.2211	-67.5842	4061	-	23.61	0.2533	1.95	2.5	1.38 ± 0.04	-	-	-	-	-

[a]Altitudes given for the river samples are subcatchment mean elevation.

[b]Analyses are normalized to ETH AMS standards S2007N (calibrated to 07KNSTD[4]) for Be, and ZAL94N (equivalent to KNSTD[5]) for Al (ref.[6]).

[c]All uncertainties are ± 1σ and include the statistical uncertainties of the AMS measurement, the blank correction (for ^{10}Be) and ICP-MS measurement uncertainty (for ^{27}Al).

Table S2 ***In situ* cosmogenic ^{14}C data.**

Sample ID	AMS ID	Extraction date[a]	Sample mass (g_{qtz})	CO_2 yield (µg)[b]	Fraction modern $F^{14}C$[c]	$\delta^{13}C$ (‰)	$^{14}C/^{12}C_{abs}$ (10^{-12})	Subtracted blank (10^5 atoms ± 1SD)	^{14}C conc. (10^5 at/g)[d]
Hilltop bedrock (*) & soil samples									
01*	48673.1.1	10/2012	4.64	44.97	2.087 ± 0.015	-19.7	2.489 ± 0.018	0.974 ± 0.351	10.26 ± 0.08
01b*	48673.2.1	02/2013	5.03	38.84	2.021 ± 0.016	-16.9	2.424 ± 0.020	0.974 ± 0.351	10.56 ± 0.11
								mean	*10.41 ± 0.14*
08	48667.1.1	10/2012	5.10	13.97	2.747 ± 0.025	-16.6	3.297 ± 0.030	0.298 ± 0.135	4.42 ± 0.05
09[e]	48676.1.1	11/2012	4.71	29.43	0.462 ± 0.006	-44.3	0.524 ± 0.005	0.974 ± 0.351	3.08 ± 0.08
	48676.1.2			29.58	0.464 ± 0.006	-37.1	0.534 ± 0.022		
13-1*	85508.1.1	02/2018	3.55	46.09	1.413 ± 0.011	-11.2	1.667 ± 0.013	0.318 ± 0.074	10.76 ± 0.09
13-3b	87892.1.1	04/2018	3.25	46.03	1.053 ± 0.009	-14.8	1.233 ± 0.010	0.318 ± 0.074	8.66 ± 0.07
13-4	86133.1.1	02/2018	3.62	49.52	1.182 ± 0.010	-16.1	1.381 ± 0.012	0.318 ± 0.074	9.38 ± 0.08
13-5	86132.1.1	02/2018	3.43	36.66	1.577 ± 0.013	-15.0	1.846 ± 0.015	0.318 ± 0.074	9.80 ± 0.08
13-7*	86131.1.1	02/2018	3.57	39.31	1.061 ± 0.010	-9.4	1.256 ± 0.011	0.318 ± 0.744	6.85 ± 0.07
15-20	87893.1.1	04/2018	3.02	34.67	0.553 ± 0.006	-9.9	0.655 ± 0.007	0.318 ± 0.744	3.67 ± 0.05
Fluvial samples									
07	48662.1.1	09/2012	5.02	21.59	1.372 ± 0.976	-17.3	1.644 ± 0.016	0.298 ± 0.135	3.45 ± 0.05
11	48661.1.1	09/2012	5.01	13.30	2.408 ± 1.020	-19.2	2.875 ± 0.029	0.298 ± 0.135	3.73 ± 0.05
12	48662.1.1	09/2012	5.00	28.16	1.089 ± 0.958	-18.6	1.301 ± 0.013	0.298 ± 0.135	3.58 ± 0.05
15-2	90400.1.1	05/2018	4.31	37.03	0.597 ± 0.006	-18.0	0.695 ± 0.007	0.310 ± 0.088	2.92 ± 0.04

[a]All samples analysed in 2012/2013 were extracted at the first ETH *in situ* ^{14}C extraction system[7], samples analysed in 2018 were extracted at the new/second ETH system[8].

[b]For samples 13-1, 13-3b, 13-4, 13-5, 13-7, 15-2, 15-20 (processed with the new ^{14}C extraction system) the CO_2 yield includes ~15 µg of ^{14}C-free CO_2, which was added as a carrier gas prior to extraction.

[c]Normalized to $\delta^{13}C$ of -25‰VPDB and AD 1950[9].

[d]All uncertainties are ± 1σ and include uncertainties related to AMS measurement and the subtracted blank.

[e]For sample 09 the gas collected was split and analysed separately by AMS on two targets. Results from both AMS analyses were added up to obtain the total ^{14}C atoms for the quartz sample. The blank was subtracted from the total ^{14}C amount, which was then divided by the sample mass to obtain the ^{14}C concentration in atoms g^{-1}.

Table S3 Apparent erosion rates calculated for ^{10}Be, ^{14}C, and ^{26}Al and their 1σ external uncertainties.

Sample ID	Apparent erosion rates (mm kyr^{-1})		
	^{10}Be	^{14}C	^{26}Al
Hilltop bedrock (*) & soil samples			
01*	2.1 ± 0.2	0.0[a]	1.8 ± 0.2
08	17.6 ± 1.5	140.0 ± 1.6	18.2 ± 1.6
09	53.1 ± 4.8	270.2 ± 23.3	121.4 ± 18.2[b]
13-1*	3.7 ± 0.3	0.0[a]	3.3 ± 0.3
13-3b	1.1 ± 0.1	11.9 ± 1.0	0.7 ± 0.1
13-4	4.9 ± 0.4	0.0[a]	3.6 ± 0.3
13-5	13.5 ± 1.1	1.9 ± 0.2	14.7 ± 1.3
13-7*	27.5 ± 2.3	28.5 ± 2.4	20.3 ± 1.9
15-20	18.7 ± 1.6	197.2 ± 16.4	22.4 ± 2.2
Fluvial samples			
07	7.2 ± 0.6	247.5 ± 20.6	9.8 ± 0.9
11	12.4 ± 1.1	202.2 ± 16.9	15.5 ± 1.4
12	7.4 ± 0.7	213.5 ± 17.8	-
15-2	20.2 ± 1.7	310.6 ± 25.9	-

[a]Measured ^{14}C concentrations are slightly higher than the steady-state, no erosion concentrations calculated for these locations.
[b]Excluded as outlier due to an implausibly low ^{26}Al concentration.

Table S4 Summary of the MCMC inversion modelling results for the 'Step change' model 1.

	Joint inversion								Individual inversion						
Percentiles	Min	5%	25%	50%	75%	95%	Max	*	Min	5%	25%	50%	75%	95%	Max
Erosion increase ($\varepsilon_2/\varepsilon_1$)															
08	19	42	52	65	95	166	260	H	7.3	40	46	56	146	559	941
09	19	35	41	49	69	115	353	H	5.7	34	43	55	89	319	781
15-20	25	77	88	107	152	261	403	H	16	76	92	126	265	762	1000
07	134	441	489	552	727	814	1000	F	99	432	479	532	642	886	1000
11	36	136	155	185	262	446	692	F	24	136	165	221	316	493	1000
12	20	290	333	387	535	910	1000	F	10	297	357	464	665	888	1000
15-2	24	185	204	230	297	491	757	F	32	191	233	348	556	831	1000
Past erosion rate ε_1 (mm kyr^{-1})															
08	4.4	5.5	6.4	6.9	7.3	7.7	15	H	1.8	3.0	4.2	6.4	7.5	8.0	23
09	4.7	10	12	14	16	18	24	H	1.1	3.8	6.9	11.8	15.9	18.2	25
15-20	3.1	3.9	4.9	5.5	5.9	6.4	19	H	0.8	1.5	2.6	5.0	6.2	6.6	15
07	0.4	0.7	1.1	1.3	1.5	1.6	4.0	F	0.3	0.4	0.6	0.9	1.2	1.5	3.9
11	1.7	2.2	2.9	3.3	3.6	3.9	13	F	0.3	0.6	0.9	1.5	2.9	3.8	14
12	0.7	1.0	1.4	1.6	1.8	2.0	25	F	0.2	0.3	0.5	0.8	1.4	1.9	23
15-2	1.3	2.0	3.1	3.6	4.2	4.6	21	F	0.3	0.5	0.8	1.8	3.7	4.7	16
Present erosion rate ε_2 (mm kyr^{-1})															
08	195	232	334	445	694	1249	1922	H	108	159	183	333	1092	4369	6960
09	322	381	533	695	1052	1868	2983	H	11	29	33	46	122	555	1050
15-20	258	306	437	578	900	1619	2497	H	149	217	245	447	1661	4973	7678
07	306	366	525	695	1078	1947	2935	F	192	304	350	409	547	1183	2112
11	260	312	450	598	930	1679	2582	F	155	218	229	255	447	1747	4394
12	269	328	472	627	974	1762	2717	F	181	238	257	286	464	1350	3410
15-2	351	437	609	800	1231	2202	3347	F	257	339	357	420	849	3279	6965
Timing of perturbation (ka BP)															
All samples	0.4	0.6	1.1	1.8	2.6	4.3	5.4								
08								H	0.1	0.2	0.7	2.6	6.8	9.4	10
09								H	0.1	0.2	0.9	3.2	6.4	9.0	10
15-20								H	0.1	0.2	0.6	2.5	6.7	9.5	10
07								F	0.5	1.0	2.4	3.7	4.8	6.2	7.9
11								F	0.2	0.5	2.6	6.5	8.6	9.8	10
12								F	0.2	0.8	2.6	5.6	7.2	8.4	10
15-2								F	0.2	0.4	1.7	4.7	7.0	8.1	8.8

[*] H=hilltop sample, F=fluvial sample

Table S5 Summary of the MCMC inversion modelling results for the 'Spike' model 2.

	Joint inversion								Individual inversion						
Percentiles	Min	5%	25%	50%	75%	95%	Max	*	Min	5%	25%	50%	75%	95%	Max
Surface lowering (m)															
08	0.6	0.7	0.8	1.0	1.0	1.1	1.3	H	0.6	0.7	0.9	1.3	1.9	2.7	3.6
09	0.8	1.0	1.3	1.7	2.0	2.5	4.6	H	0.8	1.1	1.4	2.0	2.9	4.1	5.0
15-20	0.8	0.9	1.1	1.3	1.5	1.6	1.8	H	0.8	0.9	1.2	1.6	2.2	2.9	3.3
07	1.0	1.1	1.4	1.7	1.9	2.1	2.3	F	0.9	1.1	1.3	1.5	1.8	2.1	2.3
11	0.8	0.9	1.2	1.4	1.5	1.7	2.1	F	0.7	1.0	1.2	1.5	1.9	2.5	3.3
12	0.8	1.0	1.2	1.5	1.6	1.8	2.7	F	0.9	1.0	1.2	1.4	1.7	2.1	3.9
15-2	1.1	1.2	1.7	2.2	2.5	3.0	3.3	F	1.1	1.3	1.5	1.8	2.3	3.0	3.4
Erosion rate ε_{const} (mm kyr^{-1})															
08	3.6	4.7	5.2	5.7	6.6	7.7	10	H	0.1	0.4	1.5	3.7	5.8	6.4	16
09	0.1	4.3	7.1	9.1	13	18	24	H	0.1	0.6	2.9	7.1	12	16	25
15-20	2.0	2.8	3.3	3.8	4.9	6.4	8.0	H	0.1	0.3	1.2	2.8	4.6	6.1	10
07	0.1	0.2	0.4	0.6	1.0	1.6	2.1	F	0.1	0.2	0.5	0.8	1.3	1.6	2.9
11	0.6	1.4	1.8	2.1	2.9	3.8	7.3	F	0.1	0.3	0.9	1.8	2.8	3.7	8.6
12	0.1	0.5	0.7	0.9	1.4	2.0	11	F	0.1	0.2	0.6	1.0	1.5	2.0	12
15-2	0.1	0.2	0.7	1.3	2.7	4.5	8.2	F	0.1	0.3	1.0	2.1	3.4	4.5	10
Timing of perturbation (ka BP)															
All samples	0.1	0.3	1.1	1.6	1.9	2.1	2.4								
08								H	0.1	0.4	1.5	2.7	3.6	4.2	5.0
09								H	0.1	0.3	1.1	1.8	2.3	2.7	4.3
15-20								H	0.1	0.4	1.2	2.1	2.8	3.2	4.3
07								F	0.1	0.3	0.7	1.3	1.7	2.1	2.6
11								F	0.1	0.3	1.0	1.8	2.5	2.9	3.6
12								F	0.1	0.3	0.8	1.5	2.0	2.5	3.4
15-2								F	0.1	0.2	0.7	1.3	1.7	2.0	2.4

* H=hilltop sample, F=fluvial sample

Table S6 Cosmogenic ^{10}Be-^{26}Al data and erosion rates for 18 rivers of the eastern Altiplano. Data recalculated from published data[10] using a density of 2.0 g cm^{-3} and identical production rates and scaling parameters as given in the Methods. All errors are 1σ.

Sample ID	Sampling location		Mean altitude	Measured concentrations[a]		Erosion rates[b]		Integration time	
	Lat °S	Lon °W	(m)	^{10}Be (10^5 at g^{-1})	^{26}Al (10^6 at g^{-1})	^{10}Be (mm ky^{-1})	^{26}Al (mm ky^{-1})	^{10}Be (ky)	^{26}Al (ky)
18-2	16.6437	68.3240	4022	18.30 ± 0.75	18.74 ± 1.01	14.79 ± 1.36	8.0 ± 0.8	47.9	76.9
18-4	16.5741	68.3656	4355	78.45 ± 2.36	45.69 ± 2.07	3.59 ± 0.31	3.4 ± 0.3	194.1	180.4
18-5	16.5730	68.3661	4022	36.63 ± 1.10	---	7.07 ± 0.62	---	99.4	---
18-6	16.6659	68.2869	4009	51.83 ± 1.56	37.92 ± 1.69	4.80 ± 0.42	3.5 ± 0.4	145.4	173.7
18-7	16.6832	68.2745	3915	47.65 ± 1.43	30.18 ± 1.56	5.02 ± 0.44	4.4 ± 0.4	139.4	139.8
19-1	16.9152	68.1612	4017	26.45 ± 0.80	---	10.02 ± 0.88	---	70.3	---
22-3	16.7777	68.1906	4085	15.92 ± 0.48	---	17.67 ± 1.55	---	40.1	---
22-6	17.0328	68.0941	4101	42.68 ± 1.54	28.14 ± 1.57	6.24 ± 0.56	5.3 ± 0.5	112.3	116.3
24-1	17.1060	67.9911	4172	46.00 ± 1.38	28.29 ± 1.46	5.96 ± 0.52	5.5 ± 0.6	117.5	112.7
24-2	17.1233	67.9637	4094	48.43 ± 1.45	32.98 ± 1.40	5.42 ± 0.47	4.4 ± 0.4	129.2	140.2
24-3	17.2678	67.8591	4363	13.65 ± 0.90	8.65 ± 0.69	23.63 ± 2.49	21.7 ± 2.6	30.1	28.4
24-4	17.2941	67.8099	4324	9.55 ± 0.32	8.31 ± 0.54	33.53 ± 2.98	22.2 ± 2.4	21.2	27.7
24-5	17.3840	67.6443	4214	31.17 ± 0.94	---	9.26 ± 0.81	---	76.0	---
24-6	17.3847	67.6353	4089	41.09 ± 1.23	29.85 ± 1.71	6.49 ± 0.57	4.9 ± 0.5	108.1	124.8
24-8	17.4515	67.6328	4015	25.77 ± 0.77	---	10.38 ± 0.91	---	67.9	---
25-2	17.2831	67.3090	4334	19.38 ± 0.62	12.67 ± 0.71	16.21 ± 1.43	14.3 ± 1.5	43.7	43.0
25-3	17.3304	67.3922	4177	56.87 ± 2.16	39.93 ± 2.26	4.73 ± 0.43	3.7 ± 0.4	147.6	168.0
25-4	17.3662	67.5615	4057	26.81 ± 0.81	17.60 ± 1.01	10.08 ± 0.88	8.8 ± 0.9	70.0	70.3

References

1. Knudsen, M.F. *et al.* A multi-nuclide approach to constrain landscape evolution and past erosion rates in previously glaciated terrains. *Quat. Geochronol.* **30**, 100-113 (2015).
2. Skov, D.S., Egholm, D.L., Jansen, J.D., Sandiford, M. & Knudsen, M.F. Detecting landscape transience with in situ cosmogenic 14C and 10Be. *Quat. Geochronol.* 101008 (2019).
3. Knudsen, M.F., Egholm, D.L. & Jansen, J.D. Time-integrating cosmogenic nuclide inventories under the influence of variable erosion, exposure, and sediment mixing. *Quat. Geochronol.* **51**, 110-119 (2019).
4. Nishiizumi, K., Imamura, M., Caffee, M.W., Southon, J.R., Finkel, R.C., & McAninch, J. Absolute calibration of Be-10 AMS standards. *Nucl. Instrum. Meth. B* **258**, 403-413 (2007).
5. Nishiizumi, K. Preparation of Al-26 AMS standards. *Nucl. Instrum. Meth. B* **223**, 388-392 (2004).
6. Kubik, P.W. & Christl, M. Be-10 and Al-26 measurements at the Zurich 6 MV Tandem AMS facility. *Nucl. Instrum. Meth. B* **268**, 880-883 (2010).
7. Hippe, K., Kober, F., Baur, H., Ruff, M., Wacker, L., & Wieler, R. The current performance of the in situ C-14 extraction line at ETH. *Quat. Geochronol.* **4**, 493-500 (2009).
8. Lupker, M. *et al.* In-situ cosmogenic 14C analysis at ETH Zürich: Characterization and performance of a new extraction system. *Nucl. Instrum. Meth. B* **457**, 30-36 (2019).
9. Hippe, K. & Lifton, N.A. Calculating Isotope Ratios and Nuclide Concentrations for in Situ Cosmogenic C-14 Analyses. *Radiocarbon*, **56**, 1167-1174 (2014).
10. Hippe, K. *et al.* Quantifying denudation rates and sediment storage on the eastern Altiplano, Bolivia, using cosmogenic Be-10, Al-26, and in situ C-14. *Geomorphology* **179**, 58-70 (2012).
